# Risk Perception of Health Professionals in Intrapartum Care Decisions: Protocol for a Mixed Methods Study

**DOI:** 10.2196/21443

**Published:** 2020-11-23

**Authors:** Nina H Peterwerth, Margareta Halek, Sabrina Tulka, Rainhild Schäfers

**Affiliations:** 1 Department of Applied Health Sciences - Midwifery University of Applied Sciences-Hochschule für Gesundheit Bochum Germany; 2 School of Nursing Science Faculty of Health Witten/Herdecke University Witten Germany; 3 Institute for Medical Biometry and Epidemiology Faculty of Health Witten/Herdecke University Witten Germany

**Keywords:** risk perception, decision making, obstetric health professionals, midwives, obstetricians, mixed methods, focus group discussion

## Abstract

**Background:**

Risk perception plays an important role in decision-making processes. Differences in obstetric intervention rates suggest that, in addition to medical indications, the risk perception of obstetric health professionals might have a major influence on their decision-making process during childbirth. Although studies have investigated whether risk perception affects the role of midwifery or influences decision making during childbirth, little is known about what obstetric health professionals actually perceive as risk or risky situations and whether different risk perceptions lead to more interventions during intrapartum care.

**Objective:**

The objective of this study is to understand the association of risk perception and the decision-making processes of obstetric health professionals (midwives and obstetricians) in Germany during intrapartum care. The study has 3 specific aims: (1) gain insight into what obstetric health professionals perceive as risk in the German clinical setting, (2) assess the extent to which personal and systemic factors have an impact on obstetric health professionals’ risk perception, and (3) investigate whether different perceptions of risk are associated with different decisions being made by obstetric health professionals.

**Methods:**

This is an exploratory sequential mixed methods study with 2 phases, a qualitative followed by a quantitative phase. In the first phase, qualitative data are collected and analyzed by conducting focus group discussions and applying qualitative content analysis to address aim 1. In the second phase, for aims 2 and 3 and to help explain the qualitative results, quantitative data are collected and analyzed by conducting an observational study using case vignettes within a survey constructed on the basis of the qualitative results.

**Results:**

Enrollment in the first (qualitative) phase began in July 2019, and data collection and analysis have been completed. The second (quantitative) phase is currently planned, and data collection is expected to start in December 2020. First results of the qualitative phase are expected to be submitted for publication in 2020, with completion of the second phase scheduled for 2021.

**Conclusions:**

This mixed methods study will examine the perception of risk and its association with the decision-making processes of obstetric health professionals during their care of women in childbirth. The rationale for this approach is that the qualitative data and their analysis explore participants' views in more depth, while the quantitative data will help to provide and explore a general understanding of the research problem. The results are expected to be relevant to health care professionals, policymakers, and educational institutions in order to minimize underuse, overuse, and misuse of interventions during intrapartum care.

**Trial Registration:**

German Clinical Trials Register DRKS00017172; https://tinyurl.com/y2zoowkx

**International Registered Report Identifier (IRRID):**

DERR1-10.2196/21443

## Introduction

### Background

Professionals in the field of childbirth have to make many decisions daily regarding the births they are involved in. These decisions can have far-reaching consequences for the women and children involved. They concern in particular the application and implementation of interventions during intrapartum care which have become routine in developed countries, although their effectiveness is often unproven [1]. A large number of women experience some form of intervention during labor, for example, an epidural, administration of oxytocin, an episiotomy, or a caesarean section [2,3]. However, there are remarkable major cross-national and regional differences [3-7]. For example, in 2017 the caesarean section rates in Europe ranged from 15% to 37% [8], and an even larger range of 5%-70% was found for episiotomy [8]. Clear reasons for these differences are not known [9,10]. Aside from the lack of knowledge about the effectiveness of many interventions [1,11], forensic reasons are assumed to influence the decision regarding whether or not to intervene [5,12]. Maternity care is labeled as “risk oriented” and different risk assessments are considered to be the reason for the differences in intervention rates [5]. The fear of litigation “has led to a rising level of intervention in labour” [13] and it influences health professionals’ decisions to perform interventions, for example, a caesarean section [12]. Therefore, this explains why the concept of risk and risk management has become a central principle in the care of women during childbirth [7].

MacKenzie Bryers and van Teijlingen [14] emphasize the importance of the “cultural influences, or the way risk is perceived” in provision of maternity care and refer to Downe [15], who argues that social and environmental issues such as institution, experience, and environmental impact are also important. In general, risk perception refers to subjectively perceived risk, that is, the subjectively perceived probability of the occurrence of a normally negative event [16]. Therefore, risk perception is “highly subjective” and a complex process [17]. Studies on risk perception and decision making show that nonlinear weighting of probabilities, heuristics (eg, availability or anchoring heuristics), and systematic biases play a role in risk perception [18,19]. Simplified rules of thinking and decision making lead to hasty judgments and systematic deviations between perception and reality. In addition, medium and high probabilities are often underestimated, while the occurrence of events with a lower probability are often overestimated [18,19]. Nevertheless, adequate risk perception of health professionals in maternity care is important, so that both trivialization and overestimation of risks do not lead to an inappropriate care, that is, to overuse, underuse, or misuse of interventions in labor. The significant differences in intervention rates may indicate that this is currently the case without it being possible to judge whether there is too little, too much, or adequate willingness to intervene in some regions and areas [3].

Mead and Kornbrot [20] investigated the influence of maternity units’ intrapartum intervention rates and the risk perception of midwives. The authors stated that there is a relationship between practice and perception of midwives, and that midwives working in a unit with high levels of intrapartum intervention generally had a higher perception of risk than those working in a unit with a lower intervention rate. Healy et al [21] used semistructured interviews to investigate whether midwives’ and obstetricians’ perceptions of risk affected care practices for normal birth and low-risk women in labor. They state that birth is viewed through the lens of medicalization and they consider this the reason for the routine use of interventions and technology. Researchers from the Netherlands [13,22] examined the association between a midwife’s personality, place of work, years of experience, and the timing of their decisions to make referrals from primary midwifery care to secondary obstetric care using case scenarios (vignettes). The authors found no significant correlations and stated that other factors must explain the variations in referral decisions. Nevertheless, they claim that “risk perceptions or beliefs about the course of labour influence midwives’ decisions” (p. e76). However, the transfer of these results to the German health care context is limited due to the different models of care available for women in labor.

In Germany, in general, midwives and obstetricians are involved in the care of women giving birth. According to the legal regulations, a midwife must be present at every birth and therefore all women in labor, regardless of having a low- or high-risk pregnancy, are cared for by midwives. In a low-risk birth without complications the midwife is responsible for the birth process on her own; nevertheless, in hospitals a doctor usually is also present at the actual birth. In a high-risk birth or when abnormalities occur, a doctor must be called and takes charge of the birth, but a midwife still takes care of the woman. There are some midwife-led units where women in labor with a low-risk pregnancy are cared for exclusively by midwives as long as no abnormalities occur [23], but this is rather the exception. However, most births occur in consultant-led obstetric units [2]. Therefore, women giving birth in hospitals are usually interdisciplinary cared for by midwives and obstetricians. Decisions to or not to intervene are made by a midwife alone, by a doctor alone, or together, which depend on the model of care and the circumstances. In Germany, there also are regional variations in the use of interventions. For example, the overall caesarean section rate was 30.2% in 2015 [24], but varied from 19% to 47% in western and eastern districts of Germany [5,6]. Epidurals and augmentation of labor differed significantly in hospitals with lower or higher annual number of births [25]. The high intervention rates and the regional differences lead to the assumption that, in addition to medical indications, other factors influence the decision to intervene. As other researchers showed, these might be the clinicians' personal beliefs, including the risk perception, which was an influencing factor for the choice of the mode of birth, of performing a cesarean section [12]. The association between risk perception and the decision-making processes of health professionals during intrapartum care has not yet been investigated in the German clinical setting. To gain insights into the decision making to use interventions during childbirth is of importance to study because interventions such as administering an epidural agent, intrapartum use of oxytocin, performing an episiotomy or a caesarean section are associated with short- and long term effects such as higher maternal mortality and morbidity (eg, increased risk of uterine rupture, abnormal placentation, sexual dysfunction), less satisfaction with the birthing process, a longer and more costly hospital stay, a longer recovery including the experience of pain and reduced mobility, or stillbirth, preterm birth, and greater incidence of late childhood obesity [7,26-30]. This has led to the tremendous increase in the relative cost of birth within the last century; besides, obstetric interventions during labor for women with a low-risk pregnancy, in general, are costly to the health system [31].

It is therefore crucial to gain insight into the risk perception of obstetric health professionals working in Germany so as to investigate the association between risk perception and decision-making processes in the German clinical setting in order to improve intrapartum care in Germany. Overall, in a simplified view, midwives and obstetricians are often assigned to different models of care, with midwives being assigned to the social model focusing on normality and obstetricians to the medical model focusing on technology and pathology. Although more of a continuum, this could be an indication of different approaches and perspectives due to socialization [14]. Therefore, this work focuses on the risk perception of obstetric health professionals in general, but differentiates between midwives and obstetricians to obtain more specific insights.

### Aims and Research Question

The overall aim of this study is to understand the association between risk perception and the decision-making processes of obstetric health professionals in Germany during intrapartum care. This study has 3 aims: (1) to gain insight into what midwives and obstetricians perceive as risk in the German clinical setting in order to construct valid case vignettes of risky situations; (2) to assess the extent to which personal factors (eg, age, gender, professional experience, qualification level) or systemic factors (eg, annual number of births or level of care provided, ie, secondary or tertiary) have an impact on midwives’ and obstetricians’ risk perception; and (3) to investigate whether differing perceptions of risk are associated with different decisions by midwives and obstetricians.

To this end, the research questions are as follows:

1. What do obstetric health professionals understand by risk in labor in a clinical setting, and which situations are perceived as risky intrapartum?

2. Do personal, systemic, or both factors affect the risk perception of obstetric health professionals?

3. Does risk perception affect the decisions of the obstetric health professionals or the care of women in labor?

The decisions were predefined as administering an epidural agent, intrapartum use of oxytocin, or performing an episiotomy or an unplanned caesarean section. However, adding other decisions based on the results of the first research question remained open.

## Methods

### Study Design

An exploratory sequential mixed methods design will be used to study the risk perception of midwives and obstetricians. It will first be explored via qualitative data collection and analysis. Second, a quantitative phase will be conducted involving a survey. This design has been chosen to combine the strengths of both quantitative and qualitative research [32].

In phase I the qualitative approach will be applied and narrative data collected via focus group discussions to gain an in-depth understanding of the individual risk perceptions of midwives and obstetricians in the German clinical setting and what they perceive as risky situations. The results of the focus group discussions will assist in determining case vignettes to be used in the second phase to investigate the association of different variables on risk perception and its influence on decision making. In this way, the perceptions and actions of obstetric staff will be documented and evaluated with the help of applicable and everyday case vignettes which reflect the views and personal experiences of the target population and fit the participants being studied [32]. The survey will provide the opportunity for generalization, precision, and investigation of the influence of the obstetric staffs’ risk perception on their intrapartum decision making.

The results will be reported with the Good Reporting of A Mixed Methods Study (GRAMMS) Checklist [33]. Furthermore, the Checklist for Reporting Results of Internet E-Surveys (CHERRIES) [34] is also taken into account.

### Phase I: Focus Group Discussions

A qualitative approach in the form of focus group discussions was chosen in order to conduct a preliminary exploration with midwives and obstetricians and gain insight into their views. Focus groups are not intended to “generalize […] and not to make statements about the population but to provide insights about how people in the group perceive a situation” [35]. Furthermore, focus groups are very well suited when a “range of opinions, ideas or feelings” is looked for or “when opinions or attitudes are conditional or when the area of concern relates to behaviour” [35]. Focus group discussions thus have the advantage of gaining insight into participants’ views in order to capture their voices [32,35]. The evoked discussions create synergies that could not have been achieved to this extent in a one-to-one interview [35]. In addition, group discussions are inexpensive and efficient [35].

#### Recruitment and Sampling

Midwives and obstetricians working in Germany can take part in the focus group discussions. The group composition of the contrasting or comparative groups is based on a sampling plan and on theoretically justified predefined criteria [35]. These predefined criteria have been investigated and described in several studies as possible nonclinical factors influencing clinical performance in general [36] or in the context of childbirth [12,37-41]: age, gender, number of years of experience, type of professional qualification (vocational or tertiary education), and work setting (annual number of births, level of care, care models, eg, midwife-led care/obstetrician-led care). A pool of prospective participants (stratified purposeful sample) will be generated using purposeful sampling strategies, such as typical case sampling and maximum heterogeneity sampling in order to find out which participants “can best help to understand the central phenomena” [32]. The aim will be “to capture major variations rather than to identify a common core, although the latter may also emerge in the analysis” [42]. This approach “represents less than the full maximum variation sample, but more than simple typical case sampling” [42]. Based on the topic under investigation, the predefined criteria will be taken into account to reflect the heterogeneous characteristics of obstetric professionals and, where possible, to assign participants to the different groups. Self-activation and direct contact at specialist congresses, snowball sampling, press releases, and announcements in mailing lists and on the bulletin boards of professional associations in Germany, gatekeepers, and social media platforms, such as Facebook, Twitter, and Instagram, will be used. Inclusion criteria were age (18+) and recent clinical activity: midwives employed by or affiliated with a hospital as an independent midwife; obstetrician/gynecologists employed in an obstetric department; having 1 or more years of professional experience since qualifying; and having good knowledge and understanding of the German language. Freelance midwives with no hospital affiliation or midwives who have not worked in a delivery room within the last 2 years will be excluded. According to Krueger and Casey [35], the ideal size of a focus group is between 5 and 8 participants, so 3-4 focus groups of 5-8 participants are planned.

#### Data Collection and Analysis

Krueger and Casey’s [35] recommendations will be followed such that each focus group discussion will last approximately 60 minutes (maximum 90 minutes). Further, the moderator conducting the focus group discussion will use a questioning route, developed for consistency and including opening, introductory, transition, key, and ending questions [35]. Participants will be asked to answer the following question: “What do you understand by the term ‘risk’ during labor and birth?” and “Which situation(s) in the delivery room do you consider risky?” or “Describe a situation in the delivery room that you associate risk with.”

The focus group discussions will be digitally audio recorded and the recordings transcribed using the software program f4transkript (Dr. Dresing & Pehl GmbH) [43]. Before the beginning of the discussion, participants will be asked to fill in a short questionnaire to specify demographic data (eg, age, gender, years of professional experience, annual number of births, and level of care of the past and present places of work) for the analysis process. The transcribed data will be analyzed using the software MAXQDA (version 2018.2; VERBI GmbH) according to qualitative content analysis and an analysis plan. This systematic approach includes initial examination of the data, highlighting text passages, writing notes and memos, developing codes, subcodes, and identifying an emerging code system [44,45]. The main author (NP) will be the main person responsible for the analysis process and coding, nevertheless the entire analysis process will be discussed and reflected in the research team meetings; additionally, a peer audit is planned. Discrepancies will be resolved through team discussions and reflection. The results of the focus group interviews will be used to construct case vignettes of risky situations in the delivery room. The case vignettes are intended to highlight the subjective sense of action in which the social reality of the obstetric professionals is produced. The vignettes will be checked by clinicians and experts (clinicians, psychologists, researchers) to assess their face validity in a subsequent evaluation phase and adjusted if necessary. The experts will be recruited from the professional network of the authors. The valid case vignettes will be used in phase II. In this way, qualitative findings will be used to construct the second, quantitative phase of this research project.

### Phase II: Survey

In phase II, a survey (either web-based or paper and pencil) using the case vignettes constructed in phase 1 will be used to study whether different factors such as age, gender, years of experience, annual number of births, or level of care of the clinic have an influence on the risk perception. Furthermore, the association of between the risk perceptions of obstetric health professionals and the decision-making process during intrapartum care will be investigated.

#### Recruitment and Population

Almost the same recruitment strategies and inclusion/exclusion criteria as those outlined above for phase I will be used to invite midwives and obstetricians employed in Germany to participate in the survey. The sample will be a convenience sample. Using the recruitment strategies, it is planned to invite as many midwives and obstetricians to participate as possible.

#### Data Collection and Statistical Analysis

Participants will be asked to fill out a version of either the web-based or paper-and-pencil self-constructed survey containing the vignettes of different birth scenarios. The primary endpoint is the risk perception of obstetric health professionals (midwives and obstetricians) caring for women giving birth measured on a 6-point Likert scale. The primary endpoint is thus ordinally scaled with 6 parameter values (from 0=hardly or no risk to 5=very high/highest possible risk).

As the aim is to invite as many midwives and obstetricians to participate as possible, no formal statistical sample size calculation will be carried out.

The secondary endpoint is the decision to intervene (binary with the variables “intervene yes” and “intervene no”) whereby the decisions are partly predefined as described earlier. On the basis of a 5-point Likert scale (from 1=very unlikely to 5=very likely to choose the intervention), if the score is over 3, the characteristic “intervene yes” will be assumed.

Descriptive presentation of the results of the primary endpoint (categorical; 6 categories) will be displayed through bar charts and associated absolute and relative frequencies. Other additionally recorded variables will be analyzed according to the data situation (continuous, categorical, or binary) using statistical methods appropriate to the data structure (categorial/binary: absolute and relative frequencies; continuous: medians, quartiles, means, standard deviation, minimum, maximum). Exploratory analyses for the primary endpoint (generating hypotheses) will be performed via ordinal regression using SPSS (IBM Inc) or R (R Foundation for Statistical Computing) with the following potential influencing factors: age, gender, type of professional qualification, years of experience, annual number of births or level of care of the current work location, and personality inventories. The choice of the link function to be used for this purpose will depend on the distribution of the observed cases among the different categories of the dependent variable. The quality of the model will be checked by Nagelkerke R^2^. Results of the ordinal regression will be summarized via regression coefficients, corresponding odds ratios, and by determining 95% confidence intervals for the odds ratio.

Descriptive evaluation of the secondary endpoint depending on the risk perception will be carried out using a frequency table with absolute and relative frequencies. Depending on the obtained date, either a table form with a risk perception dichotomized at cut point over 3 or a table form with a risk perception as described in the primary endpoint with 6 variables will be used.

In an additional analysis, the influence of risk perception on the decision to intervene (yes or no) will be done using logistic regression adjusted for the following variables: age, gender, type of professional qualification, years of experience, annual number of births or level of care of the current work location, and personality inventories. The choice of model is made by forward selection using a likelihood ratio test. The quality of the model will be analyzed with Nagelkerke R^2^. Results of the logistic regression are summarized with regression coefficients, corresponding odds ratios, and by determining 95% confidence intervals for the odds ratio.

Continuous data are descriptively evaluated by calculating measures of central tendency (minimum, maximum, quartiles, median, mean) and measures of variation (standard deviation, interquartile ranges, span). Graphical presentation will be made by suitable methods (eg, boxplot, scatter plot).

Categorical and binary data will be represented by calculating absolute and relative frequencies within frequency tables (potentially supported by bar charts). In addition, the risk measures (odds ratio, absolute risk) for group comparisons of interest will be calculated. An optional analysis of the actual power of the resulting models is desired via G*Power [46].

**Figure 1:**
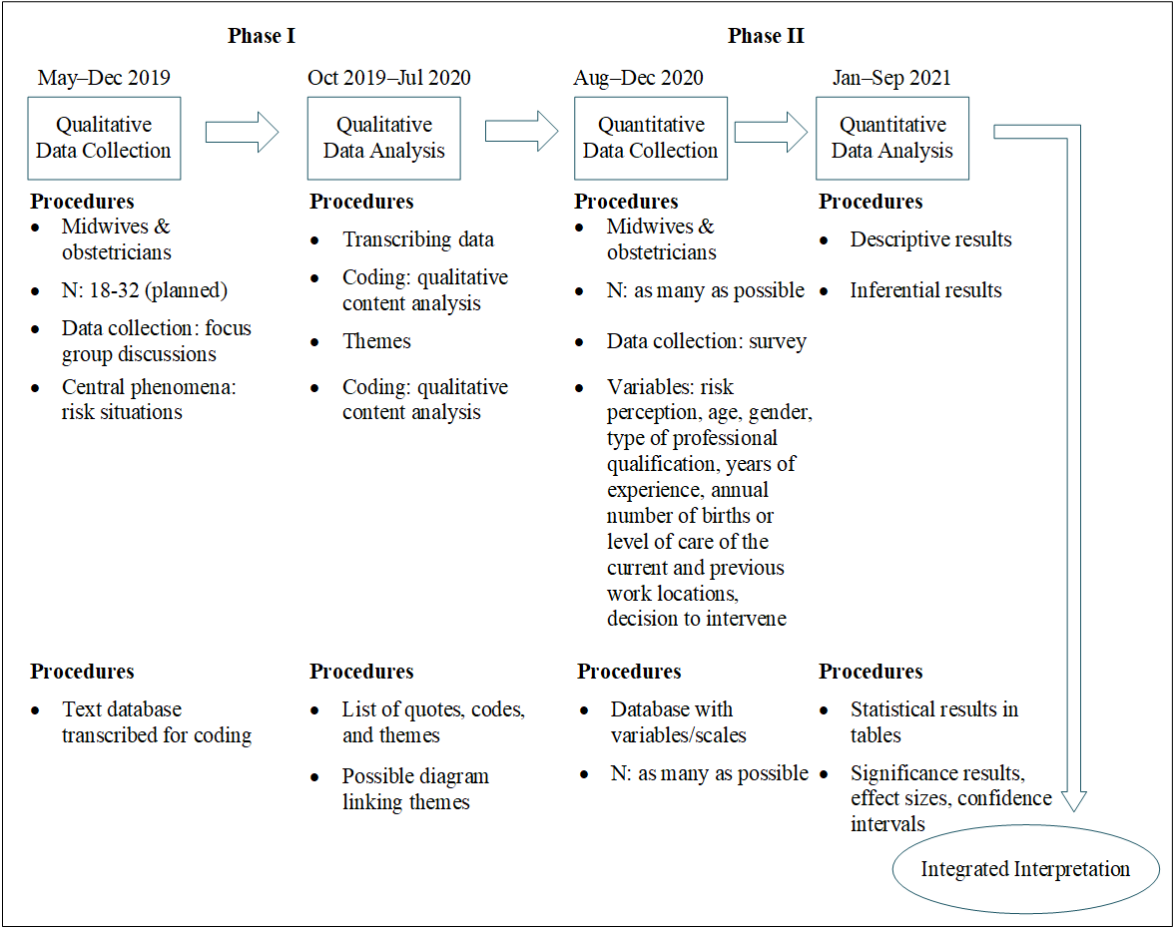
An exploratory sequential mixed methods design of this study evaluating risk perception of obstetric health professionals.

Statistical analysis will be supported by the Institute for Medical Biometry and Epidemiology at the University of Witten/Herdecke. The design and scope of the survey in phase II depend on the results of phase I, so it will be possible to adapt the content and design of the survey as appropriate. A pretest of the survey will be undertaken before fielding the questionnaire with a heterogeneous group of clinicians and research associates using a think aloud protocol and written feedback. In Figure 1 the exploratory sequential mixed methods design of this study is summarized according to Creswell [32].

### Ethics Approval and Informed Consent

The Ethics Committee of the Hochschule für Gesundheit, University of Applied Sciences, has granted ethical approval (file number 190128). Interested participants for phase I will be provided with an information sheet on the study prior to the focus group discussion and again directly before the start. Participants will have the opportunity to ask questions before they give informed consent and sign the relevant form. All participants will be informed they can withdraw from the study at any time but data can only be deleted before they have been anonymized. Privacy and confidentiality will be ensured by assigning codenames to participants and any identifying data will be changed or removed from the transcripts. Participants interested in phase II will receive information about the study, length of time of the survey, data protection regulations, and the purpose and scope of use of the data collected before starting the survey. Participants need to agree to be aware of the data protection regulations; to consent to the collection, processing, and storage of the data for the specified purpose; and to the publication of the anonymized data in written or electronic form in both versions by ticking an “I agree to participate box” at the bottom of that page or by providing written consent. Phase II responses will be collected anonymously with a certified web-based survey tool that complies with the local data protection regulations, or a printed questionnaire. All data will be securely stored/transferred to a password-encrypted computer in a locked office and will be stored securely for 10 years. Participants will not receive any incentive to participate in this study.

## Results

Participants were enrolled in the focus groups between July and September 2019. Data collection for phase I and the analysis were completed by May 2020. The results of the focus group discussions will be used to construct case vignettes of risk situations for use in phase II and for the enrollment of participants which is planned for August 2020. It is anticipated that the data collection and analysis will be completed by September 2021, and therefore results should be published by April 2022 in peer-reviewed publications.

## Discussion

### Relevance and Strength

The mixed methods design in this study is expected to provide an in-depth understanding of the risk perception of obstetric health care professionals, the extent to which risk perception is influenced by personal or systemic factors, and whether there is an association between risk perception and decision making. The mixed methods approach therefore allows researchers “to obtain a more comprehensive view” [32] about the risk perception of obstetric health care professionals than either the quantitative or qualitative perspective by capturing voices of the health professionals working in the field and investigating the association between risk perception and decision making. To the knowledge of the authors, this topic has not yet been researched in the German maternity setting, so this study may be the first to do so. The value of the mixed methods design is a “contribution of a better understanding of the problem than what might be provided by quantitative or qualitative research alone” [32].

One of the German Health Objectives (agreements between the responsible parties in the health care system) concerning childbirth is to promote physiological, low-intervention birth [47]. Thus, addressing the risk perception of midwives and obstetricians as a potential determinant influencing intrapartum decision-making processes can support the implementation and achievement of this objective. Insights into intrapartum decision-making processes and the extent to which these are affected by personal and systemic factors can help to minimize underuse, overuse, and misuse of interventions during intrapartum care. The findings might be used by care providers to give careful consideration to their decision-making process in order to modify their practice [12]. Avoiding unnecessary medical decisions can have health advantages for the women and economic advantages. The results possibly provide insight into gaps in the training of obstetric health care professionals, so that these gaps can be addressed through new training, evaluation, and reflection programs. In this way the research project outlined above supports the improvement of maternity care, and the results of this study are likely to be relevant for health care professionals, policymakers, and educational institutions in Germany and potentially internationally.

### Limitations

Case vignettes should be “a stimulating initial situation,” and encourage the participants “to make assessments or take further action” [48]. The purpose of the case vignette is to create the subjective sense of action by establishing the social reality of the obstetric staff [48]. However, a major disadvantage of vignettes “is that judgments or decisions are only hypothetical” and real assessments and actions may differ from the answers given by participants [49]. The use of case vignettes is a simplifying approach and cannot fully reflect the complexity of situations in maternity care which are influenced by verbal, visual, and intuitive information [14,49]. Nevertheless, because observations in the setting would be unethical and associated with personnel and financial challenges, case vignettes are still considered an appropriate tool in some contexts.

Distortion/bias is possible due to the recruitment methods. Self-activation using the methods described earlier may lead to the participation of particular motivated people with a special interest in the decision-making process whose response behavior differs from the overall population. This would lead to a lower variance of opinions being surveyed and there would be a possibility that the results would be influenced by the respondents and nonrespondents, respectively. Furthermore, mixed methods designs generally require more time, resources, and detailed skills in different research methods [32]. Because this research project is part of a doctoral thesis, the availability of personnel and financial resources is limited, but different strategies have been applied to address this challenge. The principal investigator (NP) is able to rely on a team of supervisors with skills and experience in different research methods. In addition, the integration into a PhD program involves continuous reflection and discussion of the methodological approaches and the consulting services of the university’s own Institute for Medical Biometry and Epidemiology. As a result, skills in quantitative and qualitative approaches are bundled together into this research project. Furthermore, this collaboration and peer audits help to deepen the researcher’s reflexivity. Because the principal investigator (NP) used to work as a midwife, her own role and experiences are continuously reflected and discussed in peer audits, both before and during data collection and analyses, in order to avoid subjective biases in interpretation.

### Conclusion

This paper outlines the methods applied in the study described above to investigate the risk perception of midwives and obstetricians and the association between risk perception and intrapartum decision making of obstetric health care professionals. The results of this study are expected to be relevant to policymakers, health care professionals, and educational institutions in order to minimize underuse, overuse, and misuse of interventions during intrapartum care.

## Abbreviations

CHERRIES: Checklist for Reporting Results of Internet E-Surveys
GRAMMS: Good Reporting of A Mixed Methods Study
